# Analytical Techniques for Characterizing Tumor-Targeted Antibody-Functionalized Nanoparticles

**DOI:** 10.3390/life14040489

**Published:** 2024-04-10

**Authors:** Ana Camila Marques, Paulo C. Costa, Sérgia Velho, Maria Helena Amaral

**Affiliations:** 1 UCIBIO—Applied Molecular Biosciences Unit, MEDTECH, Laboratory of Pharmaceutical Technology, Department of Drug Sciences, Faculty of Pharmacy, University of Porto, 4050-313 Porto, Portugal; 2 Associate Laboratory i4HB, Institute for Health and Bioeconomy, Faculty of Pharmacy, University of Porto, 4050-313 Porto, Portugal; 3 i3S—Institute for Research and Innovation in Health, University of Porto, 4200-135 Porto, Portugal; 4 IPATIMUP—Institute of Molecular Pathology and Immunology of the University of Porto, 4200-135 Porto, Portugal

**Keywords:** cancer, active targeting, functionalization, antibody-conjugated nanoparticles, electrophoresis, spectroscopy

## Abstract

The specific interaction between cell surface receptors and corresponding antibodies has driven opportunities for developing targeted cancer therapies using nanoparticle systems. It is challenging to design and develop such targeted nanomedicines using antibody ligands, as the final nanoconjugate’s specificity hinges on the cohesive functioning of its components. The multicomponent nature of antibody-conjugated nanoparticles also complicates the characterization process. Regardless of the type of nanoparticle, it is essential to perform physicochemical characterization to establish a solid foundation of knowledge and develop suitable preclinical studies. A meaningful physicochemical evaluation of antibody-conjugated nanoparticles should include determining the quantity and orientation of the antibodies, confirming the antibodies’ integrity following attachment, and assessing the immunoreactivity of the obtained nanoconjugates. In this review, the authors describe the various techniques (electrophoresis, spectroscopy, colorimetric assays, immunoassays, etc.) used to analyze the physicochemical properties of nanoparticles functionalized with antibodies and discuss the main results.

## 1. Introduction

The successful delivery of drugs to tumors is limited by systemic toxicity and is challenged by the biological barriers in the body. A promising approach to better control the traveling of anti-cancer drugs in vivo is to encapsulate them into nanoparticles that preferentially accumulate in tumor tissues, which would not only improve therapeutic outcomes but also reduce dose-limiting toxicity. While passive targeting helps localize nanoparticles in the tumor interstitium, it cannot further improve selectivity for target cells. Active targeting must step in to increase nanoparticle uptake by cancer cells, which involves decorating the nanoparticle surface with one or more types of targeting moieties (ligands) [1,2,3]. This targeted cancer therapy takes advantage of the specific interaction between ligands and surface receptors to enhance the accumulation of drug-loaded nanoparticles within cancer cells, and in turn improves overall efficacy while minimizing side effects [4]. The specificity and binding affinity of antibodies lend themselves well to the active targeting of tumors overexpressing cognate surface antigens, which function as target receptors.

The inherent complexity of antibody-functionalized nanoparticles renders them challenging to study. Hence, the characterization process should be carefully tailored to be relevant to these multicomponent nanoparticles [5]. Still, as with any other nanoparticle, a thorough analysis of their physicochemical properties is a prerequisite for any subsequent preclinical studies.

Although there have been some publications on the conjugation methods for developing these targeted nanoconjugates [6,7,8,9], little attention has been paid to the techniques used for their characterization. This review is primarily concerned with the physicochemical characterization of tumor-targeted antibody-functionalized drug-loaded nanoparticles. The authors initially provide context on nanoparticle functionalization with antibodies before delving into the techniques employed for the physicochemical characterization of these nanoparticles.

## 2. Using Antibodies to Functionalize Nanoparticles

Among the five categories of immunoglobulins (IgG, IgA, IgM, IgD, and IgE), which are classified based on their heavy-chain type, IgG is the most prevalent in human serum due to its long half-life [10,11]. The IgG molecule is a heterodimeric protein made up of two light chains and two heavy chains, with the latter being connected by a variable number of disulfide bonds in the hinge region. The structure of IgG is characterized by its Y-shape, comprising the antigen-binding fragment (Fab) region that houses the antigen-binding sites and the fragment crystallizable (Fc) region mediating effector functions [12,13].

Antibodies outperform other ligand types, such as aptamers [14,15], peptides [16,17], polysaccharides [18], and small molecules like folate [19,20], because of their distinct in vivo properties and high level of specificity [21]. The main advantages and disadvantages of the most common ligand types are listed in Table 1.

Furthermore, antibodies are abundant in free functional groups, including amine, carboxyl, and sulfhydryl, which enables them to engage in conjugation and undergo additional modifications that increase their reactivity as targeting ligands [25,26].

The repertoire of antibody ligands available for conjugation has expanded thanks to antibody fragments, such as antigen-binding fragments (Fab), single-chain variable fragments (scFv), and single-domain antibodies (sdAb). They all possess at least one antigen-binding site to ensure the function of active targeting remains. Many times, smaller antibody-derived fragments are chosen over full-length antibodies as they offer better penetration into tumors and more efficient conjugation [27,28,29].

The chemistry behind functionalization with antibodies includes physical methods, covalent methods, and avidin–biotin interaction. Those interested in learning more about the strategies for modifying the nanoparticle surface with cancer-targeting antibodies should refer to a review by Marques et al. [6]. Briefly, adsorption is a straightforward and expeditious method, but it suffers from poor reproducibility and potential detachment at off-target sites. Instead, covalent conjugation via carbodiimide-mediated coupling between amine and carboxyl groups, maleimide–thiol coupling, or click reactions is quite robust [30,31,32]. Compared to direct covalent coupling, the avidin–biotin interaction requires multi-step protocols with less efficient antibody binding [33,34].

Table 2 details the most common methods for conjugating nanoparticles with antibodies.

## 3. Characterizing Tumor-Targeted Antibody-Functionalized Nanoparticles

The unique characteristics of most solid tumors, such as vascular permeability and defective lymphatic drainage, dictate the rules for designing a nanoparticle for targeted drug delivery [36]. Nanoparticles of appropriate size (100–400 nm), shape (preferably nonspherical), and charge (preferably negative) will travel in the bloodstream for a longer time and accumulate at the tumor site by the enhanced permeability and retention effect (EPR) [37,38]. Because active targeting only occurs after passive localization of nanoparticles in the tumor, those design features are regarded as the most relevant for both tumor-targeting strategies. After conjugating the nanoparticle with the antibody ligand, a thorough characterization and comparison of the resulting conjugated and unconjugated nanoparticles should be conducted.

A rational characterization process for any biomedical nanoparticle consists of three essential components: physicochemical characterization, in vitro assays, and in vivo studies [39]. The successful application of nanoparticles in preclinical studies depends on thorough physicochemical characterization, which is required to unravel the significance of in vitro and in vivo biological data.

Given the multicomponent nature of antibody-functionalized nanoparticles, their characterization is more demanding than that of non-functionalized nanoparticles. This entails addressing additional aspects to provide a comprehensive understanding of the connection between the parts and the properties of the final nanoconjugate.

### 3.1. Conjugation Confirmation

As a first step, it is essential that nanoparticle conjugation is verified. This can be accomplished through a variety of techniques, including light scattering, microscopy, and electrophoretic and spectroscopy techniques, all of which will be discussed below.

#### 3.1.1. Light-Scattering Techniques

Any characterization should consider the examination of particle size and surface charge, yet these analyses can also give some important clues to the effectiveness of the coupling reaction. Light scattering-based techniques, namely dynamic light scattering (DLS) and electrophoretic mobility, are used to estimate hydrodynamic size and zeta potential based on fluctuations in the intensity of light scattered by dissolved or suspended particles [40]. Along with an increase in mean particle size, it is common for the zeta potential values to decrease [41,42], increase [43,44,45], or even shift [46] as a result of functionalization. For instance, when conjugating gemcitabine-loaded chitosan nanoparticles with sialic acid and cetuximab (CTX), i.e., a monoclonal antibody (mAb) directed against the epidermal growth factor receptor (EGFR), Kumar et al. [42] noticed a reduction in zeta potential due to electrostatic interactions between cationic chitosan and anionic ligands. In contrast, the decoration of lenvatinib-containing poly (lactic-co-glycolic acid) (PLGA) nanoparticles with CTX using carbodiimide chemistry resulted in less negatively charged particles arising from the neutralization of PLGA’s carboxylate anionic groups [44]. In another work [45], the zeta potential of polystyrene nanoparticles increased from −45.3 ± 1.1 mV to −25.1 ± 7.2 mV after coupling with anti-CD44v6 half-antibody through maleimide chemistry, given the N-terminal amine groups in the antibody fragment. There can be cases, however, where functionalization is not accompanied by significant changes in surface charge, as in the development of calcium phosphosilicate nanoparticles attached to anti-CD71 via carboxy-polyethylene glycol (cPEG) [47].

#### 3.1.2. Microscopy Techniques

Although light-scattering techniques have several advantages, such as ease of sample preparation and quick measurements, the premise that all nanoparticles are homogeneous and spherical can lead to inaccuracies in the calculated dimensions [48,49]. This limitation has been addressed by conducting morphological studies that rely heavily on microscopy. Observing antibody-conjugated nanoparticles in the range of millimeters to nanometers can give physical, chemical, and structural information that is closely related to their performance [50]. Therefore, microscopy-based methods, including scanning electron microscopy (SEM), transmission electron microscopy (TEM), or atomic force microscopy (AFM), are recommended as a complement to size data obtained from DLS measurements. With these techniques, a better grasp of the shape, roughness, agglomeration, and elemental composition (purity) of nanoparticles can be obtained [51]. As well as providing insights into shape transformation during functionalization, all three techniques can image and measure dried samples of different-sized nanoparticles, contrary to DLS. Nevertheless, their physical basis, contrast formation mechanism, and maximum achievable resolution vary, as does the performance. SEM is generally considered to be as accurate as the other two techniques for larger particles but less suitable for small nanoparticles. Moreover, while TEM offers the highest throughput, AFM images have lower noise levels and better resolution. Even though the most appropriate technique still depends on the sample type and the information sought, selection is often driven by the availability and familiarity with the equipment [52].

**Figure 1:**
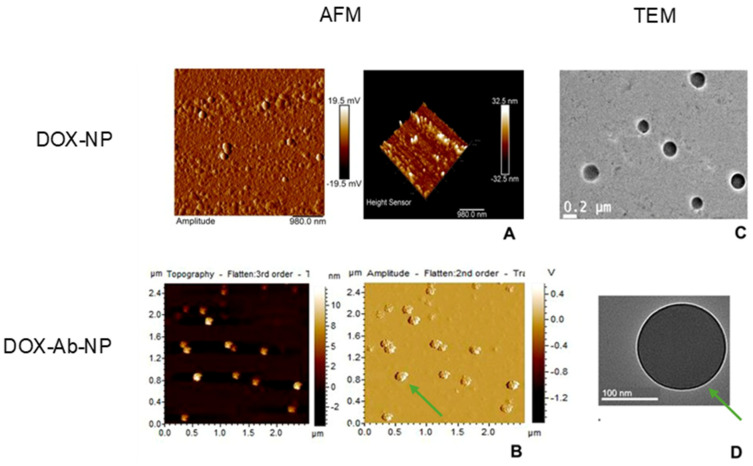
AFM (**A**,**B**) and TEM (**C**,**D**) images of untargeted doxorubicin-loaded nanoparticles (DOX-NPs) and CD340-targeted doxorubicin-loaded nanoparticles (DOX-Ab-NPs). The rough surface (**B**) and intense bright shadow around the particle (**D**), denoted by green arrows, indicate the presence of the antibody on the nanoparticle surface. Adapted from [56] under the terms of the Creative Commons Attribution—Non-Commercial (unported, v3.0) License.

Using TEM, Yang et al. [53] visualized the conjugation of liposomes with the antibody fragment as a change in color contrast, with the targeting ligands appearing as a grayish shell around a darker core. Depending on the antibody-conjugated nanoparticle, this can also be seen as a darker coating on its surface compared to the unconjugated nanoparticles [54]. It is possible, however, that this type of microscopy may not be useful for verifying functionalization if antibody ligands introduce no visible structural alterations. As described by Revilla et al. [44], the development of CTX-conjugated PLGA nanoparticles containing lenvatinib proved successful, but was undetectable by TEM analysis. Occasionally, researchers have undertaken more than one microscopy analysis to gather information on antibody-conjugated nanoparticle morphology. For example, in developing CD44-conjugated mesoporous silica nanoparticles, the diameter difference between the bare and conjugated nanoparticles determined by SEM matched the results from AFM measurements [55]. The increased height of mesoporous silica nanoparticles upon antibody coupling confirmed successful attachment to the surface. In Figure 1, the AFM 3D analysis showed that the surface of CD340-targeted doxorubicin (DOX)-loaded nanoparticles were no longer smooth upon coverage with an antibody directed against C340 (or human epidermal growth factor receptor 2, HER2) [56]. Also, in this study, TEM images revealed a bright shadow surrounding the particle, not observed with the untargeted DOX-loaded nanoparticles, hence the presence of the antibody previously labeled with fluorescein isothiocyanate (FITC) (Figure 1).

Alternatively, Wang et al. [57] added a FITC-labeled rabbit anti-human IgG to unconjugated and CTX-conjugated solid lipid nanoparticles (SLNs) encapsulating DOX to perform fluorescence microscopy. As expected, only the conjugated SLNs exhibited green fluorescence from FITC after the secondary antibody was bound to the ligand. Having used the same technique, Abdolahpour et al. [58] also identified green dots as DOX-loaded nanostructured lipid carriers (NLCs) coated with an anti-EGFRvIII mAb. Also known as nanoscopy, super-resolution optical microscopy has brought fluorescence microscopy to the nanoscale, and in recent years, its applications have gradually expanded to include nanoparticle characterization [59,60]. By eliminating conventional ensemble averaging, super-resolution microscopy enables characterizing nanoparticle heterogeneity on a single-particle basis using fluorescence labeling. While super-resolution techniques are still underemployed in conjugated nanoparticle imaging, they have already opened new avenues for studying antibody orientation (See Section 3.2.2).

#### 3.1.3. Electrophoretic Techniques

With its ability to separate proteins based on their molecular weight, gel electrophoresis, such as sodium dodecyl sulfate (SDS)–polyacrylamide gel electrophoresis (PAGE), has been used for determining whether antibody conjugation has been successful. Before loading onto the PAGE gel, proteins are usually treated with the anionic detergent SDS, resulting in negatively charged complexes. Their negative charge, and thus their migration through the gel in an electric field, is proportional to the relative size of the polypeptide chain [61]. After being separated by SDS-PAGE, the proteins are stained with, for example, Coomassie brilliant blue dye (Bradford reagent) to visualize the corresponding bands in the gel [62].

**Figure 2:**
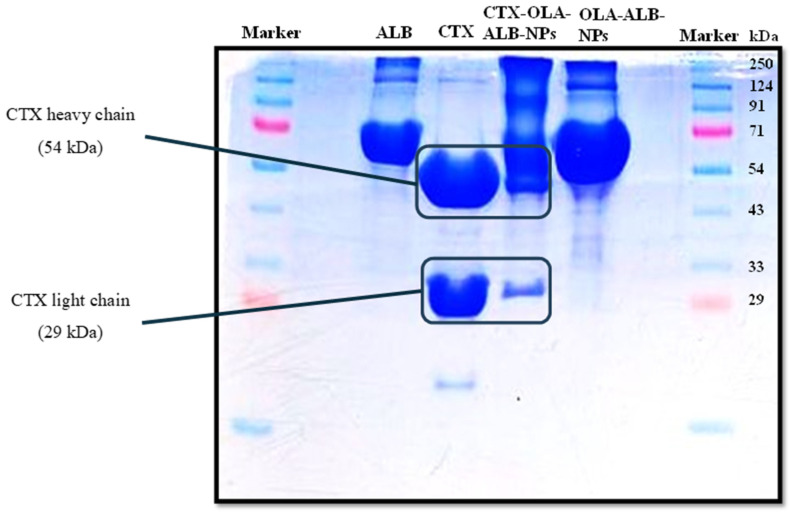
SDS-PAGE of pure albumin (ALB), pure cetuximab (CTX), cetuximab-functionalized oleanolic acid-loaded albumin nanoparticles (CTX-OLA-ALB-NPs), and non-functionalized oleanolic acid-loaded albumin nanoparticles (OLA-ALB-NPs). Adapted from [66], copyright (2023), with permission from Elsevier.

Peng et al. [63] examined the apparent molecular weight of trastuzumab (TZM) or herceptin-conjugated, paclitaxel-loaded worm-like nanocrystal micelles by SDS-PAGE. The smeared band could only be seen in the image of the conjugated micelles, not in the two controls (herceptin). This was most likely the result of an increase in the number of TZM antibodies conjugated with these micelles. This electrophoretic technique also demonstrated the decoration of liposomal triptolide with half-antibodies against the carbonic anhydrase IX, which caused the band to be displaced upward as the molecular weight changed [64]. In another work [65], SDS-PAGE was performed on free rituximab, rituximab-coated NLCs, and the supernatant obtained after the physically coated NLC dispersions were centrifuged. The partial displacement of the conjugated NLC band was linked to the successful rituximab conjugation, as the protein became heavier and thus more difficult to move in the electrophoretic field. The surface attachment of CTX to oleanolic acid-loaded albumin nanoparticles was better understood by SDS-PAGE analysis of pure albumin, CTX, unconjugated nanoparticles, and conjugated nanoparticles [66]. The latter showed two distinct bands, one corresponding to albumin and the other matching one of the antibody’s characteristic bands (Figure 2).

**Figure 3:**
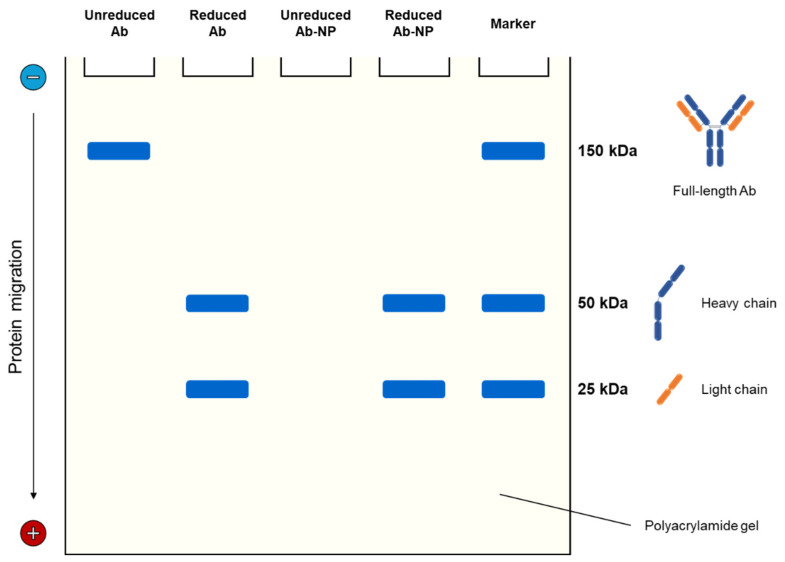
Schematic representation of reducing and nonreducing SDS-PAGE analyses of nanoparticle conjugation with IgG-like antibodies via maleimide chemistry. The antibody (Ab) is made up of two identical heavy (blue) and light (orange) chains linked by disulfide bonds. The two heavy chains are connected by disulfide bonds in the hinge region. In the reduced Ab lane, two bands at 50 kDa and 25 kDa are visible, corresponding to the Ab heavy and light chains, respectively. As for antibody-conjugated nanoparticles (Ab-NP), Ab can only be released from nanoparticles that have been reduced. No Ab release is observed without reduction (unreduced Ab-NP lane). These observations altogether confirm successful conjugation via thioether linkage.

Regarding maleimide chemistry, thioether linkages can only be formed between maleimide-activated nanoparticles and antibody free sulfhydryl groups, which can be either introduced into an intact antibody by thiolation or obtained by selectively reducing its disulfide bonds, yielding two monovalent halves [67]. Figure 3 presents an SDS-PAGE analysis of nanoparticle conjugation with antibodies via maleimide chemistry.

Keeping that in mind, Khanna et al. [68] evaluated antibody-functionalized PLGA nanoparticles by SDS-PAGE under reducing and nonreducing conditions to confirm the formation of thiol–maleimide bonds. Given that the release of half-antibodies only happened after the reducing step, the nanoparticles were assumed to be covalently conjugated with the antibodies primarily via their heavy chains. Similar results were seen when the coupling of paclitaxel-loaded PLGA nanoparticles with thiolated antibodies against the TNF-α transmembrane form was investigated using nonreducing and reducing SDS/PAGE [69]. In the case of cubosomes as paclitaxel carriers, anti-EGFR Fab’ conjugation via maleimide chemistry also increased the effective molecular weight of the sample, resulting in upward migration of the band under nonreducing conditions [70]. Following treatment with a strong reducing agent (dithiothreitol), the band of conjugated cubosomes corresponding to the heavy chain rather than the light chain migrated upward, suggesting that conjugation occurred at the hinge region of the Fab’.

Although some authors performed SDS-PAGE for this characterization, its widespread application is limited by labor-intensive and time-consuming procedures for gel preparation [71].

#### 3.1.4. Spectroscopy Techniques

The spectroscopic identification of the bond formed between antibody and nanoparticle is an improved way of confirming antibody coupling, which has been done widely by Fourier-transform infrared (FTIR) spectroscopy.

Covalent conjugation of anti-TRAIL (CD253) mAb with oxaliplatin-loaded SLNs using 1-ethyl-3-(-3-dimethylaminopropyl) carbodiimide (EDC) as a cross-linker led to the emergence of the amide bond at 1651 cm^−1^ and amine stretching at 3500 cm^−1^ in the FTIR spectrum [72]. For targeted delivery of diallyl disulfide to triple-negative breast cancer cells, Siddhartha et al. [73] successfully modified the SLN surface with an antibody directed to the receptor for advanced glycation end products (RAGE) via carbodiimide chemistry, as evidenced by a stronger band at 3507.47 cm^−1^ the authors ascribed to an aromatic secondary amine group. Related to the S-S bond, the appearance of a peak at 549 cm^−1^ helped to confirm the functionalization of mesoporous silica nanoparticles with TZM through maleimide chemistry, as already suggested by the zeta potential measurements [74]. Instead of FTIR, nuclear magnetic resonance (NMR) spectroscopy and Raman spectroscopy can also be utilized for determining the functional groups. They all operate through different mechanisms, endowing them with quite a few differences. Of note, FTIR enables quick analysis of samples other than aqueous solutions because of the intense infrared absorption of water. While Raman is more sensitive to functional groups than FTIR, the spectra take longer to be acquired and are susceptible to interference from fluorescent samples. NMR can detect polar molecules with low background noise, which are frequently associated with weak Raman signals [75,76].

In the case of conjugating the antibody ligand before nanoparticle formation (pre-conjugation), the antibody-conjugated material is examined spectroscopically rather than the antibody–nanoparticle conjugate. As an example, Silveira et al. [77] proceeded to manufacture nanoparticles after the ^1^H NMR spectrum of the final polymeric conjugate had revealed the characteristic peaks of MFE-23 scFv (δ = 8.11, 7.95, 7.22, 6.81 and 6.61 ppm) and maleimide-terminated PEGylated PLGA (δ = 5.20, 4.91, 3.50 and 1.46 ppm), indicating that the antibody fragment was coupled with the targeted polymer. Among spectroscopic techniques, X-ray photoelectron spectroscopy (XPS) benefits from chemical specificity and surface sensitivity up to a depth of <10 nm, delivering quantitative information about the elemental composition of nanoparticle near surface regions [78]. Knowing the considerable amount of nitrogen groups in antibody molecules, the coupling between a mAb directed against programmed death ligand 1 (PD-L1) and polyethylene glycol–poly(ε-caprolactone) was verified using XPS by the presence of a distinct signal peak from the 1s orbital of nitrogen (N 1s) that was absent in the control spectrum [79]. According to the XPS spectra of CTX-decorated and undecorated chitosan nanoparticles, the low intensity peak in the N 1s binding region prior to conjugation was replaced by a strong N 1s peak due to the additional nitrogen from CTX [80]. Because the signal peak associated with nitrogen was only visible in the XPS spectrum of CD147-targeted oxidized dextran, Tian et al. [81] were able to demonstrate that the Schiff base reaction between anti-CD147 mAb amine groups and oxidized dextran aldehyde groups took place.

**Figure 4:**
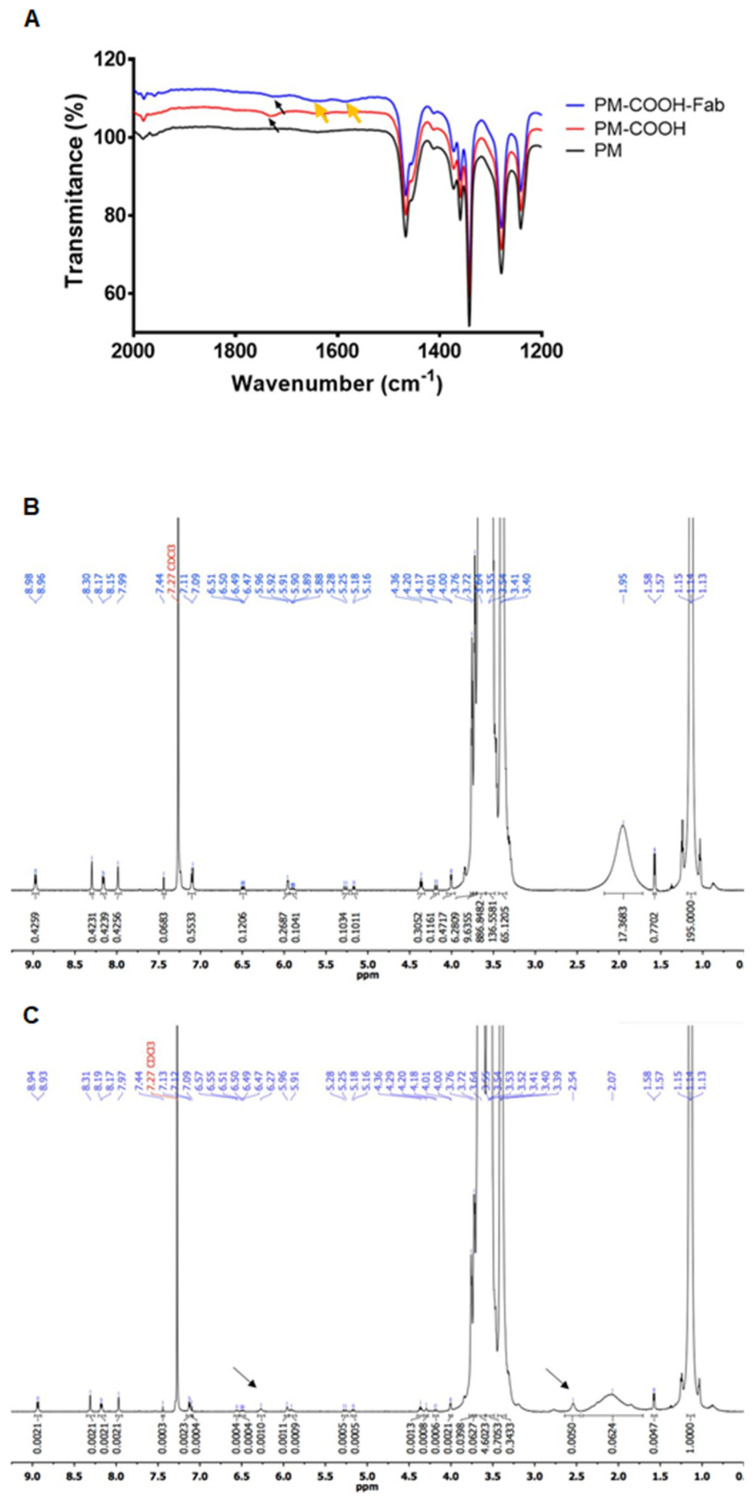
FTIR spectra (**A**) of unconjugated polymeric micelles (PM), carboxylated polymeric micelles (PM COOH), and Fab-CD44v6-conjugated polymeric micelles (PM-COOH-Fab); ^1^H-NMR spectra of niclosamide-loaded polymeric micelles (**B**) and Fab-CD44v6-conjugated niclosamide-loaded polymeric micelles (**C**). Adapted from [82], copyright (2021), with permission from Elsevier.

To gain a deeper chemical understanding of antibody conjugation, some authors subjected the developed nanoparticles to various spectroscopic analyses. To illustrate, FTIR and ^1^H NMR analyses were carried out to confirm the conjugation of Fab-CD44v6 with polymeric micelles [82]. Summarizing the spectroscopic data in Figure 4, an amide bond was formed (Figure 4A, yellow arrows), and new peaks at δ = 6.27 and 2.54 ppm (Figure 4C, black arrows), representing the olefin structure and protons from the antibody fragment, stood out in the ^1^H NMR spectrum of the conjugated micelles. In a study by Raju et al. [83], the occurrence of a N 1s signal peak at 398.7 eV and amide bond stretching vibrations (3300 and 1630 cm^−1^) in the FTIR spectrum was attributed to the antibody (TZM) and its conjugation with tocopheryl polyethylene glycol succinate (TPGS) liposomes via carbodiimide chemistry, respectively. Zhu et al. [84] performed Raman spectroscopy and XPS during the characterization of SiO_2_-coated inorganic nanoparticles modified with bevacizumab. The three new Raman peaks appearing at 891 cm^−1^, 965 cm^−1^ and 1122 cm^−1^ were associated with secondary amides, primary amides, and the C–C-OH structure of the antibody. Together with the peak from the C-S group in the XPS spectrum, which was related to bevacizumab, these findings suggested that covalent binding using N-hydroxysuccinimide (NHS)/EDC was achieved.

A little differently, Khaleseh et al. [85] concluded that TZM-conjugated liposomes (immunoliposomes) had been formed based on FTIR and differential scanning calorimetry (DSC) findings, namely, the observation of the endothermic peak of TZM in the thermogram of the immunoliposomes.

### 3.2. Antibody Quantification and Orientation

In addition to selecting the appropriate target cell receptor and antibody ligand, ligand density (i.e., the number of ligand molecules per nanoparticle) and their orientation are two other critical factors in maximizing the performance of active targeting [86].

#### 3.2.1. Quantification of Antibodies Bound to Nanoparticles

In theory, higher antibody density increases the chances of antibody-conjugated nanoparticles binding to the target receptor by virtue of multivalency and avidity. However, overcrowded nanoparticles with closely packed antibodies do have the following weaknesses: (i) the stealth properties are reduced, (ii) the nanoparticle hydrodynamic radius increases, preventing deeper penetration into the tumor, (iii) cellular uptake decreases due to high receptor occupancy, and (iv) steric hindrance is present, which increases when the ligands face the same direction [87].

Researchers benefit from measuring the antibody content because it allows them to predict the efficacy of antibody-conjugated nanoparticles in vivo and helps control the coupling protocol, ensuring consistency and reproducibility during manufacturing. The quantification of antibodies can be expressed as the amount of antibody per nanoparticle and is often converted into the number of antibodies attached. Alternatively, it can be presented as conjugation efficiency, which is calculated as the percentage ratio of antibody quantified (directly or indirectly) in the nanoconjugate to the total antibody initially employed for conjugation.

Antibody quantification has been widely documented by colorimetric protein assays, particularly Bradford and bicinchoninic acid (BCA) assays. Whereas the Bradford assay relies on the binding of the Coomassie brilliant blue dye to proteins, the BCA assay is based on the ability of peptide bonds to reduce Cu^2+^ to Cu^1+^ and form purple complexes [88]. The BCA assay was used by Khanna et al. [68] to estimate the antibody content on PLGA nanoparticles, yielding a result of 9 ± 4 μg antibody/mg nanoparticles, with approximately 40 antibody molecules per particle. The amount of TZM and panitumumab Fab fragments conjugated to polymeric nanoparticles as determined by the Bradford assay was around 13 μg Fab per milligram of nanoparticles, corresponding to 12 ligands per nanoparticle [89].

It is possible to determine antibody concentration directly by combining antibody-conjugated nanoparticles with BCA or Bradford reagents and measuring absorbance at 562 and 595 nm, respectively. Herein, the final nanoconjugate is often purified first with Sepharose CL-4B columns to remove any unbound antibodies [90,91,92]. On the other hand, the indirect method involves measuring the unconjugated antibody in the supernatant collected after centrifuging antibody-conjugated nanoparticle suspensions/dispersions at the end of the conjugation process. The calculated free antibody in the supernatants is then deducted from the total antibody used. In one study, the total T-cell-receptor-mimicking scFv coupled with liposomes was calculated from Lowry–Peterson colorimetric assay values [93].

Notwithstanding the utility of colorimetric techniques, the amino acid composition and glycosylation of the antibody can interfere with the assay responses [94]. Fluorescent techniques emerged as an alternative to overcome the limitations that such bias can impose. Following the production and purification of targeted liposomal cisplatin, the uncoupled OX26 mAb was measured using a fluorescence method with NanoOrange™ (Invitrogen, Carlsbad, CA, USA), a merocyanine dye [95]. In a direct approach, Domínguez-Ríos et al. [96] conjugated PLGA nanoparticles with TZM tagged with fluorescein for antibody quantification by fluorescence spectrophotometry.

Of note, in the case of coupling biotinylated CB11 mAb with streptavidin-conjugated SLNs, the extent of mAb binding to biotin and subsequently to the SLN surface could not be quantified directly [97]. Instead, the optimal amount of biotinylated antibody and streptavidin was determined by monitoring the average particle size and zeta potential of the obtained SLN–streptavidin–antibody complexes.

Aside from being a critical aspect of nanoconjugate characterization, conjugation efficiency is also an important parameter to consider when optimizing the antibody/lipid ratio in immunoliposomes [98,99]. The antibody/nanoparticle ratio [100], as well as the conjugation method and type of antibody ligand (full length or its fragments), all have an impact on conjugation efficiency. Varshosaz et al. [101] produced NLCs with three different fatty amines and decorated the nanocarriers with TZM using physical and chemical methods. Regardless of the fatty amine used in NLC manufacturing, adsorption was better with TZM conjugation than maleimide chemistry as per the Bradford method. Compared to the intact antibody, antibody fragments grant benefits to the conjugated nanoparticle, namely, increased levels of conjugation. As an example, Duan et al. [102] modified PEG-PLGA nanoparticles with TZM and TZM-derived Fab’ and achieved a coupling efficiency of 27.7 ± 1.67% and 64.8 ± 2.32%, respectively, using the BCA assay.

Table 3 summarizes the antibody quantification method and conjugation efficiency described in some papers on the development of antibody-conjugated nanoparticles for cancer therapy.

According to the data in Table 1, there is a general trend toward higher conjugation efficiencies with indirect methods. This raises the possibility that indirect BCA and Bradford assays may overestimate the level of antibodies in the supernatants. In this way, direct methods appear to be more accurate in measuring surface ligand density. Furthermore, unless the functionalized nanoparticles are analyzed at the single-particle level, any significant disparities in ligand density between particles will go unnoticed when assessing multiple particles concurrently. Flow cytometry is a sophisticated technique that enables multiparametric analysis of individual particles [108]. As such, Rodallec et al. [109] developed a quantitative method based on flow cytometry and prototyped “IgHk calibrator beads” to determine the exact number of coated antibodies per nanoparticle. In a model of HER2^+^ breast cancer, this new method was successfully applied to establish the optimal number of TZM molecules to be grafted onto liposomes for maximum effectiveness. Integrating sizing and fluorescence analysis, the method adopted by Chen et al. [110] to quantify different targeting moieties on the surface of liposomes was based on nanoflow cytometry, with ligand density being derived from particle size and the number of ligands on the same nanoparticle.

#### 3.2.2. Control of the Orientation of Attached Antibodies

Depending on the conjugation method, antibodies can be oriented or randomly immobilized on the nanoparticle surface. Due to random immobilization in physical adsorption and carbodiimide chemistry, proper antibody orientation cannot be guaranteed. Through antibody modification, conjugation protocols based on maleimide chemistry and click chemistry ensure immobilization in an oriented manner. However, while selective reduction in the antibody’s disulfide bonds yields site-specific free sulfhydryl groups, lysine residues modified with thiols (thiolation) are randomly distributed over the antibody. Because of this drop in site selectivity, thiolation results in heterogeneous conjugates and may affect their antigen-binding capacity [67]. If possible, antibodies should be attached to nanoparticles through the Fc region, leaving the antigen-binding sites in the Fab region free to recognize and interact with the target antigen (receptor). Adapter molecules like biotin and avidin (or their derivatives) can also be employed to achieve site-specific conjugation [111]. Recent strategies using Fc-binding proteins and metal ions through coordination bonding, rather than chemical modification of antibodies, also afforded effective control over their orientation [31,112]. In a study by Brückner et al. [113], both the conjugation chemistry and the amount of functionalized antibody on the nanoparticle surface were found to be significant factors in the spatial orientation of the covalently linked antibodies. Also, non-directional coupling of antibodies on planar surfaces mainly resulted in the ideal “end-on” orientation (Fc region attached to the surface) at high densities, whereas “side-on”-oriented antibodies (Fab and Fc region attached to the surface) were more prevalent at low densities [114].

As much as the in vitro evaluation of functionalized nanoparticles for targeting capacity and cytotoxicity can provide indirect insight into Fab accessibility, a proper investigation of antibody orientation is seldom performed, even for conjugation chemistries that occur randomly. There is also a need for more research on antibody accessibility in pre-conjugated nanoparticles, since antibodies may not have an outward orientation. At the same time, direct methods for monitoring antibody orientation on nanoparticles are lacking. One way of studying antibody orientation is to use a fluorophore-labeled secondary antibody to recognize the available Fab region in primary antibodies coupled with nanoparticles [115]. More recently, different super-resolution methods based on direct stochastic optical reconstruction microscopy (dSTORM) [116], spectrally resolved direct stochastic optical reconstruction microscopy (SR-dSTORM) [117], and DNA point accumulation for imaging nanotopography (DNA PAINT) [118] have been developed to elucidate the orientation of the antibodies at the single-nanoparticle level. Woythe et al. [116] employed dSTORM to determine the number of accessible (i.e., functional) CTX antibodies covalently conjugated with silica nanoparticles, with the targeting interaction (CTX-EGFR) serving as a labeling tool. In subsequent work [117], SR-dSTORM allowed for the simultaneous quantification of total and functional CTX antibodies conjugated with silica nanoparticles. The functionality of CTX-conjugated nanoparticles was observed to vary when the antibody concentration was altered. Tholen et al. [118] expanded the toolbox of super-resolution microscopy by using Fc-targeting and Fab-targeting probes conjugated with single-stranded DNA to quantify and map both Fc and Fab antibody domains on the nanoparticle surface.

### 3.3. Biological Activity of Antibody-Functionalized Nanoparticles

Understanding the biological activity of antibody-functionalized nanoparticles requires a thorough analysis of their interactions with target receptors using 2D and 3D cell cultures as in vitro and in vivo preclinical models, which is beyond the scope of this review. Still, before proceeding to the evaluation in cells, tissues and animal models, antibody integrity after functionalization and immunoreactivity of the obtained nanoconjugates can be assessed.

#### 3.3.1. Confirmation of Antibody Integrity after Functionalization

The process of conjugation may disrupt the structure of antibodies, resulting in diminished or altered bioactivity. To ensure that the conjugated antibodies retain their biological activity, it is important to assess their integrity following attachment to nanoparticles. Different conjugated antibodies have been evaluated for integrity using SDS-PAGE.

By comparing the electrophoretic migration profiles of free anti-CD44v6 and anti-CD44v6-functionalized SLN to that of the unbound ligand collected by ultrafiltration/centrifugation, Cavaco et al. [119] found that antibody integrity was preserved during conjugation. Bevacizumab integrity following NLC functionalization was verified using reducing SDS-PAGE [90]. As anticipated, two characteristic bands corresponding to the heavy and light chains of bevacizumab were visualized at 48 and 25 kDa, indicating that the antibody was coupled intactly with docetaxel-loaded NLC. In a work by Eloy et al. [120], TZM exhibited two bands (50 kDa and 25 kDa) on electrophoresis gel under reducing conditions, representing its heavy and light chains, respectively. This suggested that TZM was attached intact to the liposomal surface, similar to the observations of Sakhi et al. [121], who developed paclitaxel-loaded PLGA nanoparticles with TZM coating. Likewise, the evaluation of TZM integrity on other immunoliposomes by SDS-PAGE revealed that the antibody did not undergo degradation, as evidenced by the presence of two distinct bands at 25 kDa and 50 kDa [91].

**Figure 5:**
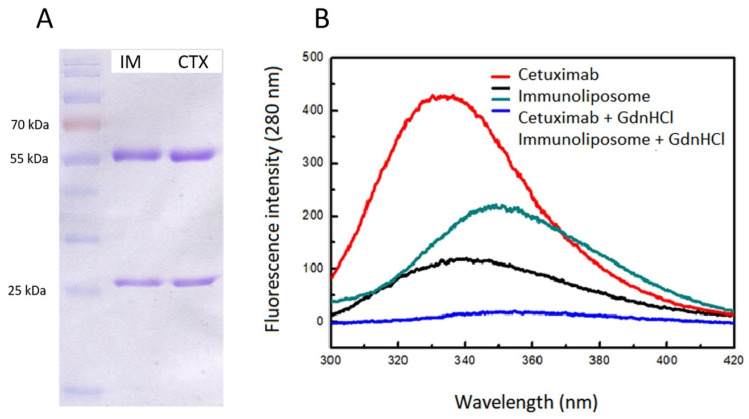
Assessment of cetuximab (CTX) integrity by (**A**) SDS-PAGE analysis of immunoliposomes (IM) and free CTX and (**B**) fluorescence spectroscopy of cetuximab and immunoliposomes, with or without incubation with guanidine hydrochloride (GdnHCl) denaturant. Precision Plus Protein Dual Color Standard (Bio Rad, Hercules, CA, USA) was used as the protein size marker on the SDS-PAGE gel. Reprinted from [92], copyright (2020) with permission from Elsevier.

Fluorescence spectroscopy using intrinsic probes (tryptophans and tyrosines) provided complementary information on CTX integrity to that obtained by SDS-PAGE [92]. Based on the electrophoretic profiles of immunoliposomes and CTX depicted in Figure 5, it can be noted that the antibody was not damaged or had its primary structural integrity impaired, as evidenced by the two bands at 55 kDa and 25 kDa, corresponding to the antibody heavy and light chains, respectively. According to fluorescence emission spectra at an excitation wavelength of 280 nm (Figure 5B), in which tryptophans and tyrosines were excited, CTX had a maximum emission wavelength (λ_max_) at 336 ± 1 nm. Incubating CTX and immunoliposomes with guanidine hydrochloride denaturant resulted in a slight red shift (λ_max_ at 350 nm and 340 nm, respectively) and fluorescence suppression, indicating exposure of the primary fluorophore—tryptophans. These observations altogether suggested that the local tertiary structure of CTX was somewhat altered by functionalization with liposomes, but denaturation did not occur.

Peng et al. [63] took a different approach, employing circular dichroism (CD) spectroscopy to further investigate any conformational changes in herceptin (TZM) conjugated with micelles. This optical spectroscopic technique capitalizes on the distinct absorption of left- and right-circularly polarized light by chromophores to derive structural information about protein conformations [122]. The CD spectra showed that TZM’s secondary structure transitioned from a beta-turn to a polyproline II helix conformation upon conjugation with micelles [63].

#### 3.3.2. Evaluation of the Immunoreactivity of Antibody-Functionalized Nanoparticles

To remain specific, antibodies must be able to recognize and bind to specific antigens after conjugation. If the ligand functionality is lost, antibody-functionalized nanoparticles will be ineffective in drug delivery to target cells.

As immunoassays rely on specific antibody–antigen reactions, indirect enzyme-linked immunosorbent assay (ELISA) is often used for evaluating nanoconjugate immunoreactivity. The general procedure for performing this assay begins with coating the plate with the antibody ligand’s cognate antigen, then washing and adding a blocking buffer. Following rewashing, the developed antibody-functionalized nanoparticles are introduced and incubated. Indirect ELISA involves an additional step of adding an enzyme-linked secondary antibody complementary to the primary antibody. Horseradish peroxidase is one of the most common substrates that causes a blue color change. Typically, the optical density (OD) is measured at 450 nm and plotted against antibody concentration [123].

**Figure 6:**
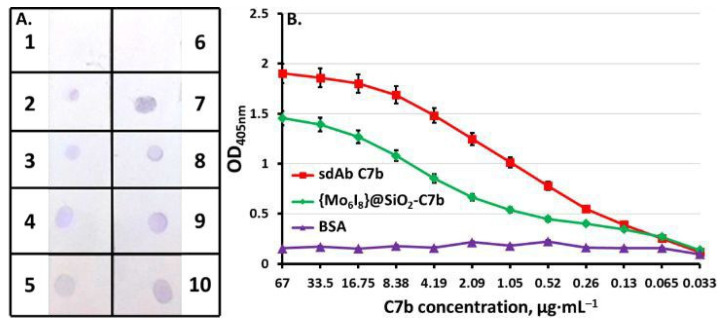
(**A**) Qualitative analysis of the activity of single-domain antibody (sdAb) C7b and its nanoconjugates by dot-blot assay: 1, 6—bovine serum albumin (BSA) (negative control); 2, 7—C7b-conjugated silica nanoparticles (3.4 µg and 6.7 µg of C7b, respectively); 3, 8—sdAb C7b (3.4 µg and 6.7 µg, respectively); 4, 9—secondary antibody control; 5, 10—conjugate control. (**B**) Quantitative assessment of the activity of C7b and the conjugated silica nanoparticles ({Mo6I8}@SiO_2_-C7b) by ELISA. Reprinted from [126], copyright (2020) with permission from The Royal Society of Chemistry.

Through indirect ELISA, Abdolahpour et al. [58] investigated the immunoreactivity of an anti-EGFRvIII mAb conjugated with DOX-loaded NLC and found that it remained unchanged despite conjugation. This group utilized a similar conjugation process in a subsequent work, connecting anti-EGFRvIII mAb to curcumin-loaded PLGA nanoparticles, and attained the same outcomes in terms of immunoreactivity as determined by ELISA [124]. The ability of OX26-modified PEGylated liposomal cisplatin to bind to the transferrin receptor was also studied using ELISA [95]. The results demonstrated that the targeted liposomal formulation had a significantly higher absorbance at 405 nm than the non-targeted formulation, implying that the OX26 retained its bioactivity after coupling to liposomes. In one study by Narayanaswamy et al. [125], this immunoassay also confirmed the specific activity of mAb 2C5 attached to the liposome surface via a polymeric linker. Slightly more informative was the analysis by Vorotnikov et al. [126], who combined dot-blot assay and ELISA to obtain both qualitative and quantitative data (Figure 6) on the activity of HER2-specific sdAb C7b alone or conjugated with silica nanoparticles.

Looking at Figure 6A, all samples stained the nitrocellulose paper onto which recombinant human ErbB2/HER2 was absorbed, indicating a specific interaction with the target antigen. As a result, it can be concluded that the antibody produced is active and has maintained a high level of specificity following the conjugation process. These results were consistent with the data from ELISA (Figure 6B), which demonstrated an effective interaction between sdAb C7b and HER2/neu at a concentration up to 0.065 µg/mL. The same was true for nanoparticles conjugated with C7b, despite a 25% reduction in affinity for the nanoconjugate, probably related to the irregular orientation of sdAb C7b on the nanoparticle surface and in turn less accessibility to the antigen [126]. This leads us to the view that evaluating the immunoreactivity of antibody-conjugated nanoparticles also sheds light on the orientation of the attached antibodies.

## 4. Conclusions

The development of tumor-targeted antibody-functionalized nanoparticles is a notably more challenging process than that of non-targeted nanoparticles, and their characterization presents an additional layer of complexity as well. This is because the parts of these multicomponent systems (nanoparticle and antibody ligands) must work in concert to achieve specificity.

In vitro and in vivo studies are paramount to elucidate the biological activity of antibody-functionalized nanoparticles regarding biocompatibility, cytotoxicity, safety, and efficacy. However, prior to preclinical development, researchers should collect comprehensive data on the physicochemical and structural characteristics of the obtained nanoconjugates.

Depending on the aspect evaluated, different techniques may be available, each with its own advantages and disadvantages. In practice, choosing a technique is not only influenced by the features of the sample but also by its ease of execution and equipment availability.

While most authors already confirm functionalization and quantify the coupled antibody, the orientation and integrity of antibody ligands, as well as the immunoreactivity of nanoconjugates, are often overlooked. The fact that the current methods for monitoring antibody orientation, for example, are scarce and somewhat complex may be contributing to the lack of such analysis. Notwithstanding the great progress made with immunoassays and several methods based on spectroscopy and electrophoresis, there is still room for further refinement and integration of other technologies, such as flow cytometry, into more frequent use. Soon, nanoparticle characterization will rely more heavily on artificial intelligence. This advanced tool has already assisted novel immunological approaches using multiplex assays and high-throughput platforms, which enable simultaneous detection of multiple antigens and antibodies [127]. Additionally, machine learning may help predict the immunogenicity of the nanoconjugates obtained, as well as the interactions between the antibody-conjugated nanoparticles and the target antigen [128,129].

The characterization of antibody-functionalized nanoparticles will continue to improve and become more precise as more sensitive methods are developed and implemented.

## Figures and Tables

**Table 1:** The main advantages and disadvantages of different cancer-targeting ligands used for nanoparticle conjugation.

Ligand Type	Advantages	Disadvantages	Ref.
Antibodies	High affinity and specificity	Large size, poor tissue penetration Risk of immunogenicity Expensive	[22,23]
Aptamers	Small size, little impact on nanoconjugate size Broad range of target recognition Little or no immunogenicity	Conjugation can affect their proper folding, 3D structure, and binding affinity	[14]
Peptides	Moderate size, better tissue penetration Less immunogenicity	Lower binding affinity Susceptibility to digestion by protease	[24]
Small molecules	Small size High stability Non-immunogenicity Low cost	Low specificity, off-target toxicity	[20,23]

**Table 2:** A summary of the most used methods for nanoparticle conjugation with antibodies [6,7,30,35].

Conjugation Type	Coupling Method	Functional Groups	Bonds	Orientation
Non-covalent methods	Physical adsorption	Various	Hydrogen, hydrophobic interactions, van der Waals forces	Random
	Ionic adsorption	Charged	Electrostatic interactions	Oriented
Covalent methods	Carbodiimide chemistry	Carboxyl Amine	Amide	Random
	Maleimide chemistry	Sulfhydryl Amine	Thioether	Oriented (Ab thiolation), site-specific (Ab selective reduction)
	“Click” chemistry	Azide, alkyne (CuAAC) Azide, cycloalkyne (SPAAC) Tetrazine, strained alkene (iEDDA)	Triazole (CuAAC, SPAAC) Pyridazine (iEDDA)	Site-specific
Use of adapter molecules	Avidin–biotin interaction	Negatively charged Sulfhydryl or carbohydrate	Multiple hydrogen and hydrophobic interactions (K_d_: 4 × 10^–14^ M)	Mostly oriented, site-specific (Fc-biotinylation)

Ab: antibody; CuAAC: copper (I)-catalyzed azide–alkyne cycloaddition; Fc: fragment crystallizable; iEDDA: inverse electron demand Diels–Alder; K_d_: dissociation constant; SPAAC: strain-promoted azide–alkyne cycloaddition.

**Table 3:** Quantification of antibodies on the surface of tumor-targeted nanoparticles.

Quantification Method	Antibody Ligand	Nanoparticles	Conjugation Method	Conjugation Efficiency	Ref.
Direct BCA	Bevacizumab	NLC	Maleimide	62%	[90]
Direct BCA	Trastuzumab	Liposome	Maleimide	62.78%	[91]
Direct BCA	Cetuximab	Liposome	Maleimide	53.3%	[92]
Indirect BCA	Cetuximab	Liposome	Maleimide	~94%	[103]
Indirect BCA	CD44 Ab	Liposome	Maleimide	79.5 ± 2.9%	[104]
Indirect BCA	CD56 Ab	PLGA-PEG	Maleimide	84.39 ± 1.01%	[105]
Indirect microBCA	Cetuximab	PLGA	Carbodiimide	76%	[44]
Direct Bradford	TRAIL mAb	SLN	Carbodiimide	52%	[72]
Direct Bradford	Rituximab	NLC	Physical adsorption	89 ± 0.15%	[65]
Indirect Bradford	sLeA mAb	PLGA	Carbodiimide	67 ± 3.0%	[106]
Indirect Bradford	Trastuzumab	PLGA	Carbodiimide	~63%	[107]
Indirect Bradford	Trastuzumab	PCL-PEG nanocrystal micelles	Carbon-nitrogen	52.6%	[63]

Ab: antibody; BCA: bicinchoninic acid; CD: cluster of differentiation; NLC: nanostructured lipid carrier; mAb: monoclonal antibody; PEG: polyethylene glycol; PCL: poly(ε-caprolactone); PLGA: poly(lactic-co-glycolic acid); sLeA: sialyl Lewis A; SLN: solid lipid nanoparticle; TRAIL: tumor necrosis factor-related apoptosis-inducing ligand.

## Data Availability

Not applicable.
